# Climate-Induced Elevational Range Shifts and Increase in Plant Species Richness in a Himalayan Biodiversity Epicentre

**DOI:** 10.1371/journal.pone.0057103

**Published:** 2013-02-20

**Authors:** Yasmeen Telwala, Barry W. Brook, Kumar Manish, Maharaj K. Pandit

**Affiliations:** 1 Department of Environmental Studies and Centre for Interdisciplinary Studies of Mountain and Hill Environment, University of Delhi, Delhi, India; 2 School of Earth and Environmental Sciences and Environment Institute, University of Adelaide, Adelaide, Australia; 3 University Scholars Programme, National University of Singapore, Singapore, Singapore; University of Western Australia, Australia

## Abstract

Global average temperature increase during the last century has induced species geographic range shifts and extinctions. Montane floras, in particular, are highly sensitive to climate change and mountains serve as suitable observation sites for tracing climate-induced biological response. The Himalaya constitute an important global biodiversity hotspot, yet studies on species’ response to climate change from this region are lacking. Here we use historical (1849–50) and the recent (2007–2010) data on temperature and endemic species’ elevational ranges to perform a correlative study in the two alpine valleys of Sikkim. We show that the ongoing warming in the alpine Sikkim Himalaya has transformed the plant assemblages. This study lends support to the hypothesis that changing climate is causing species distribution changes. We provide first evidence of warmer winters in the region compared to the last two centuries, with mean temperatures of the warmest and the coldest months may have increased by 0.76±0.25°C and 3.65±2°C, respectively. Warming-driven geographical range shifts were recorded in 87% of 124 endemic plant species studied in the region; upper range extensions of species have resulted in increased species richness in the upper alpine zone, compared to the 19^th^ century. We recorded a shift of 23–998 m in species’ upper elevation limit and a mean upward displacement rate of 27.53±22.04 m/decade in the present study. We infer that the present-day plant assemblages and community structure in the Himalaya is substantially different from the last century and is, therefore, in a state of flux under the impact of warming. The continued trend of warming is likely to result in ongoing elevational range contractions and eventually, species extinctions, particularly at mountaintops.

## Introduction

Global average surface temperature has increased by approximately 0.6–0.8°C during the last century [1], [2]. Average maximum temperature (during the period 1950–2000) for Sikkim, as interpolated from Worldclim data [3], is 8.1°C, varying from −10.9°C to 28°C (±8.3°C). The average minimum temperature is 0.25°C, varying from −23.4°C to 18.1°C (±7.6°C). According to the CCMA B2A warming scenario, annual mean temperature of Sikkim is likely to increase by 1.82°C in 2050 and 1.93°C for 2080. For 2050, average minimum and maximum temperatures are projected to increase by 0.8°C and 2.8°C, respectively and for 2080 by 0.93°C and 2.95°C, respectively. Numerous studies have shown that climate warming has induced species’ geographic range shifts and occasional extinctions [4]–[6]. The warming induced upward shifts of species occur along latitudinal [7] and altitudinal [8] clines. A number of studies have suggested alpine plants to have evolved adaptations in response to the long-term climate change from the Pleistocene to late Holocene epochs [9], [10]. Low temperature is considered as the main controlling factor of alpine plant life in a mountain ecosystem [11], [12]. Therefore, montane floras, in particular, are highly sensitive to climate change since their distribution is mainly controlled by climate related ecological factors that dominate at high altitudes. Mountains are thus, regarded as particularly suitable observation sites for tracing climate-change impacts [13], [14]. The Himalayan gradient represents the longest bioclimatic elevational gradient and constitute the highest elevational limits of vascular plant species (6400 m) on Earth [15]; these montane ecosystems are undergoing rapid warming, increase in precipitation, and extensions of the growing season [16]. Alpine plant diversity of the Himalaya is higher than the global average [13] and significantly higher plant diversity and richness is found between 4200 and 4500 m elevations compared to similar elevations of any other mountain range [17].

The collision between the Indian and the Eurasian plates in early Eocene (soft collision) and Miocene (hard collision) resulted in the formation of the Himalaya [18], [19]. The phase-wise evolution of the Himalaya over 45–60 million year (myr) period provided novel opportunities to the floral and faunal elements, arriving from all directions, to colonize the newly evolving landscapes and subsequently diversifying into unique ecosystems (see [20], [21], M.K. Pandit and V. Kumar, unpublished data). This youngest major mountain range of the world, houses many endemic biotas with low resilience [22] and is vulnerable to climate change [23]. Yet the region remains neglected and unrepresented in scientific literature on species’ response to the changing regional and global climate [24]. This study provides the first time analysis of plant species’ response to climate change in the most sensitive alpine areas of the Himalaya using historical and new field data.

Eastern Himalaya was particularly chosen as a study site due to a number of reasons; there is a general lack of scientific studies in the region. This region also faces a high threat of terrestrial biodiversity loss due to landuse change, deforestation and infrastructure development [25]. Eastern Himalaya exhibit a high degree of endemism in alpine plants compared to the Western Himalaya and other neighboring regions [26]. Alpine meadows are among the 200 critical global ecoregions [27]. Previous work has suggested that most of the endemic alpine plant species in this region are highly habitat specific [28]. Yet the region is increasingly exposed to large-scale land-use change and has suffered high biotic extinctions over the last few decades [25], [26].

Given this context, the key questions we address here are: (i) do plant species in the Eastern Himalaya show elevational range shifts when compared to their historical distributional records, (ii) is there any change in the species richness and composition of the mountain summits, and (iii) have any species been extirpated during the last century from the alpine areas of this region under the impact of warming?

## Methods

### Site Description

**Figure 1:**
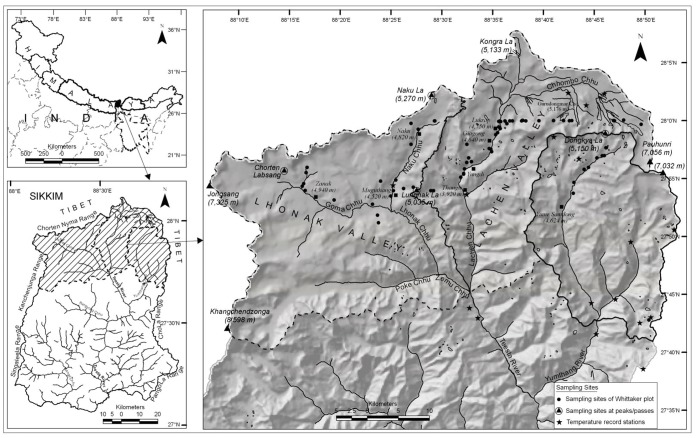
Map of the study area. Vegetation sampling locations (circles), four mountains passes (triangles) whose uppermost 200 m elevations were studied for plant species richness, and the locations of the temperature recording stations (stars).

We investigated two alpine valleys namely, Lachen and Lhonakh. Lachen, spread over an area of 700 km^2^ is situated between 27°45′00′′–28°7′53′′N Latitude and 88°26′53′′–88°0′54′′E Longitude. Lhonakh, spread over 991 km^2^ is situated between 27°40′15′′–28°03′13″ N Latitude and 88°7′09″–88°32′21″ E Longitude. Both are located in the eastern Indian state of Sikkim, which forms a part of the Eastern Himalaya (Figure 1). The total geographic area of Sikkim is 7096 km^2^ and nearly 60% of this Indian state comprises temperate and alpine areas above 3000 m elevation [29]. The two valleys studied possess highly heterogeneous habitats with rapid changes in geology, orientation, climate and vegetation along the altitudinal cline [29], [30]. Highest altitudinal limits of alpine plant species in Sikkim are found in Lachen and Lhonakh valleys. We identified various alpine communities in the Lachen and Lhonakh valleys based on dominant vegetation type. We calculated geographic areas of each of the three elevational zones (4000–4500 m, 4500–4800 m and 4800–5500 m) in Lachen and Lhonakh valleys with the help of Digital Elevation Models (DEMs) using ArcGIS ver. 9.1.

### Temperature Data

The historical ambient-air-temperature data for the study area in the form of mean diurnal range, mean of the warmest and the coldest month and temperature lapse rate (the rate of decrease of temperature with increasing elevation) were taken from secondary sources [30]. For recent data we recorded daily minimum and maximum ambient-air temperature for four years (2007–2010) at 17 stations located within the study area (Figure 1). Even though a single data point represents the 19th century historical temperature record, we considered this record to be of high importance and the only basis for calculating variations with reference to our recent observations. The amount of change our results represent are consistent with the regional analysis of temperature shifts detected in the recent analysis [16]. We considered the difference between historical and recent temperature data significant only if the former fell outside the average range ± S.D of the latter.

### Species’ Elevational Ranges

The historic data of plant species’ distribution ranges for Lachen and Lhonakh valleys were sourced from published records [30], [31]. Of all the species reported from the alpine areas of these valleys, we selected only 124 endemic species whose exact locations of occurrence and elevational ranges were known from the historic records (see Table S1). We were cautious not to include sedges and grasses because these comprise major part of the feed of grazers and the distributional changes in these groups are affected by vegetative propagation of rhizomes/runners under the influence of grazing. However, we wish to clarify that neither any grazing was observed during our field visits nor was there any published record of grazing in the study region. Of the 124 endemic plant species selected, 93 inhabited the lower elevational band of the alpine zone (4000–4500 m), 15 species were in the intermediate elevational zone (4500–4800 m) and 16 species were found to occur within the highest elevational zone (4800–5500 m). However, only 7 and 14 species showed exclusive distribution in the lower, intermediate and the highest elevation zones, during historical and recent time periods, respectively. However 113 and 110 species showed varying degrees of overlap between these three elevational bands during historical and recent records, respectively.

In addition, a list of all the species known to occur during 19^th^ century at the uppermost 200 m elevational band of the four mountain passes located within the study area, i.e., Dongkya la, Lungna la, Naku la and Chortenima la, was compiled from the published records [30]–[32]. This list of species was also verified from the herbarium records available at CAL (Botanical Survey of India, Central National Herbarium, Kolkata) and SHC (Botanical Survey of India Sikkim Circle, Gangtok). The historical botanical accounts of previous expeditions [30] were of particular importance because of the temperature data recorded by the authors for different altitudes within the study area for 1849–50.

The recent surveys were carried out in 2007–2010 during July and August (the same months as mentioned in the historical records). Necessary permissions were obtained from all the relevant Departments of Government of Sikkim (Letter No. GOS/H-II/97/56(Part)/314 dated 1.06.2005; Letter No. 31/P&S/GOS/FEWMD dated 27.07.2005; Letter No. 77/SO/F dated 05.04.2008 & Letter No. 66/SRO/F dated 15.07.2009). A nested modified Whittaker plot design was used for vegetation sampling and elevation range mapping [33]. We modified the plot study for mapping the species’ ranges, as each Whittaker plot covers a large area. In this study each plot covered an area of 1000 m^2^ and contained nested subplots of three sizes: one 100 m^2^, two 10 m^2^ and ten 1 m^2^ located within the main plot. The nested subplots increased the probability of detection of even the locally rare species. A line transect along altitudinal cline of 4000–5550 m in each of the two valleys was surveyed and sampled. Plots were laid along the transect after every 50–100 m rise in elevation, covering all the habitat types. Plant species were recorded in 1000 m^2^ area in each plot. Frequency, density and basal cover of all the plant species in the ten 1 m^2^ and two 10 m^2^ subplots were also recorded. The geographic location of all the plots was recorded using GPS (Magellan TM, SporTrack Map). In total, 55 plots were laid in the two valleys (34 plots in Lachen and 21 in Lhonakh).

The altitude along the transect at which a plant species was recorded for the first and last time in the Nested modified Whittaker plot were marked as its recent lower and upper range margins, respectively. In this manner the distribution ranges of all the 124 endemic species were recorded and designated as recent elevational records. This exercise was repeated consecutively for three years to improve sampling effort and minimize the chances of error in recording the elevational distribution margins.

The uppermost 200 m elevational bands of each mountain pass (referred to above) were surveyed in detail for plant species present there and all the species were recorded. Locating and recording species at these passes was not difficult because of their limited number that ranged from 3 to 31. The region is largely undisturbed due to absence of anthropogenic activities; all the passes studied experienced little, or no direct human disturbance, and grazing by domestic livestock was absent. We assume that the present-day plant distributions in these remote high-altitude habitats are not significantly impacted by biotic pressure (beyond those that exist within the community, such as interspecific interactions of the resident species), therefore, changes represent a natural progression. Also, the historical records of species are assumed to reflect true accounts of their diversity and distributions because the species were too common to be overlooked by the previous authors.

### Data Analysis

#### Temperature change

From the mid-19^th^ century historical records, the mean temperature of the warmest month, of the coldest month and mean diurnal range were obtained for three altitudes: 3352 m, 4572 m, and 5791 m in the two valleys [30]. For comparative analysis of the early 21^st^ century situation, we established 17 meteorological stations in these valleys at three altitudinal bands: (i) 3200–3500 m (5), (ii) 4400–4600 m (6), and (iii) 5300–5555 m (6). The temperatures at all these stations were recorded daily during survey period of 2007–2010. For each station, average daily minimum and maximum temperatures for the coldest (February) and the warmest (August) months were calculated. The average temperature values recorded for all the stations located within similar altitudinal belt gave the mean temperature of the coldest month for that particular elevational band. Mean diurnal range difference between daily minimum and maximum temperatures was calculated and then averaged for each month. The temperature lapse rate was calculated by analyzing the change in temperature after every 100 m ascent in elevation.

#### Species’ ranges and richness

Recent species’ range margins were compared with the historical records of the selected 124 endemic species and the changes, if any, were estimated. The difference of ≤100 m in the values of recent and historic range margins was ignored, irrespective of positive or negative shift, to account for sampling errors during the two time periods. Species’ mid-altitudinal ranges (mean of the lowermost and the uppermost altitudinal range) for the two time periods were calculated for all species. The difference in the mid-altitudinal range of a species between historical and recent records was used to calculate the median shift shown by the species (with percentile confidence intervals derived from 10,000 bootstrap re-samples).

Species richness patterns across the elevational gradient at an altitudinal interval of 100 m were plotted for all the 124 species using the historic and the recent elevational range extent data. These richness patterns depicted the shifts, if any, in species richness maxima [34]. In addition, the changes in species richness at the uppermost 200 m elevational band at four mountain passes were also compared. Increase or decrease in species’ number was estimated for each of the four mountain passes; absence of a historically reported species was regarded as its loss or probable extinction from that area and presence of a species not reported historically was taken to be a new arrival.

To enable a meaningful comparison between the study conducted in Alps and current study conducted in the Himalaya with respect to upward shift in species’ upper elevational limit, we followed the methods outlined in previous studies (see [35]). Out of 124 endemic species, we considered only those species (n = 96) which showed greater than 20 m upward shift from their historical upper elevational limit. Differences between present and historical upper elevational limits were recorded to document species’ upward shift.

The same dataset (n = 96) was then used for calculation of mean upward species displacement rates/decade, i.e. species’ mean upward migration rate. Migration rate was computed as follows (see [36]):
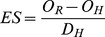
where, *ES = *elevational shift per decade*; O_R_ = *present uppermost elevation limit of species*; O_H_ = *historical uppermost elevation limit of species; D_H_ = number of decades since historical investigation (i.e. ∼10 decades for present study).

ES values obtained for each species was then averaged over the entire dataset (n = 96) to obtain mean upward species displacement rates/decade.

## Results

### Temperature Trends

The comparison between the historical (1850) and the recent (2007–2010) ambient air temperature data revealed a warming trend in the study area during the last century (see Table S2). The mean temperature of the warmest and the coldest months increased by 0.76±0.25°C and 3.65±2°C, respectively. The changes in daily maximum and minimum temperatures between the two time periods resulted in a narrowing of diurnal temperature range (DTR) by an average of 3°C. The difference between the two time periods was most significant for the higher elevational band of 4400–4600 m compared to the lower bands. The lapse rate appears to have reduced from 0.82°C/100 m in 1850 to 0.42°C/100 m in 2007–2010. Notably, the results showed more warming in winters than in summers, with the differences in mean temperatures of the coldest and the warmest months during the century being 3.75°C and 0.5°C, respectively. At higher elevations (5300–5500 m) the changes in the mean temperatures of the coldest and the warmest months during the last ∼150 years were 5.61°C and 0.8°C, respectively (see Figure S1). The lower altitudinal band (3200–3500 m) recorded a change of 1.61°C and 1.0°C in the mean temperatures of the coldest and the warmest months, respectively.

### Species’ Range Shifts

Sikkim Himalaya region has nearly 4250 plant species [32] with almost a third of this diversity being found above 4000 m elevations. The areas above 4000 m in the Lachen and Lhonakh valleys harbor about 350 vascular plant species, of which nearly 60% and 32% are endemic to the Himalaya and Eastern Himalaya, respectively. Our endemic species dataset (n = 124) represent nearly 50% of the alpine plant families from Eastern Himalaya; 70% of the families and 33% of the all the species occurring in Lhonakh and Lachen valleys. We recorded a total of 31 families comprising 92 herb and 32 shrub species. The life form spectra in our endemic species dataset (n = 124) revealed dominance of hemicryptophytes in all the three elevational zones (36.8% in both 4000–4500 m and 4500–4800 m; 41.8% in 4800–5500 m). Hemicryptophytes were followed by geophytes in all the three elevational zones (32.2% in 4000–4500 m; 29.8% in 4500–4800 m; 29.7% in 4800–5500 m).

The areas in north Sikkim are characterized by three dominant alpine community types: dwarf scrub (4000–4500 m), meadows (4500–4800 m) and steppe (4800–5500 m). For the Lachen and Lhonakh valleys, the areas representing the three elevational zones (4000–4500 m, 4500–4800 m and 4800–5500 m) are 6412, 10126 and 51972 hectares, respectively.

**Figure 2:**
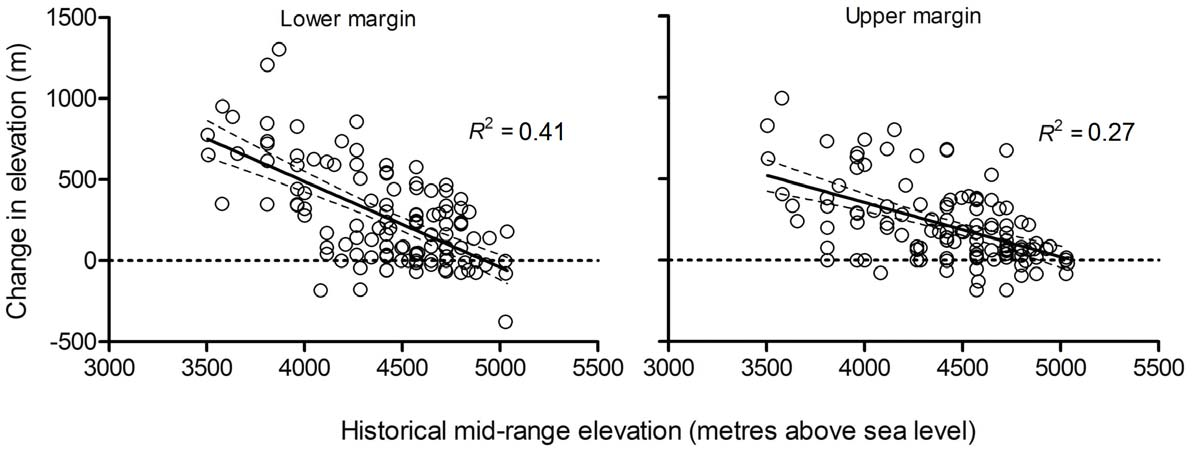
Change in the elevational range margins for endemic plant species during the last century. Change in elevation of the lower (left panel) and upper (right panel) range margins for 124 endemic plant species between historical (1850–1909) and recent (2007–2010) periods. The dashed horizontal line represents zero net shift. A linear regression line (and 95% confidence intervals) is also shown for each subplot.

**Figure 3:**
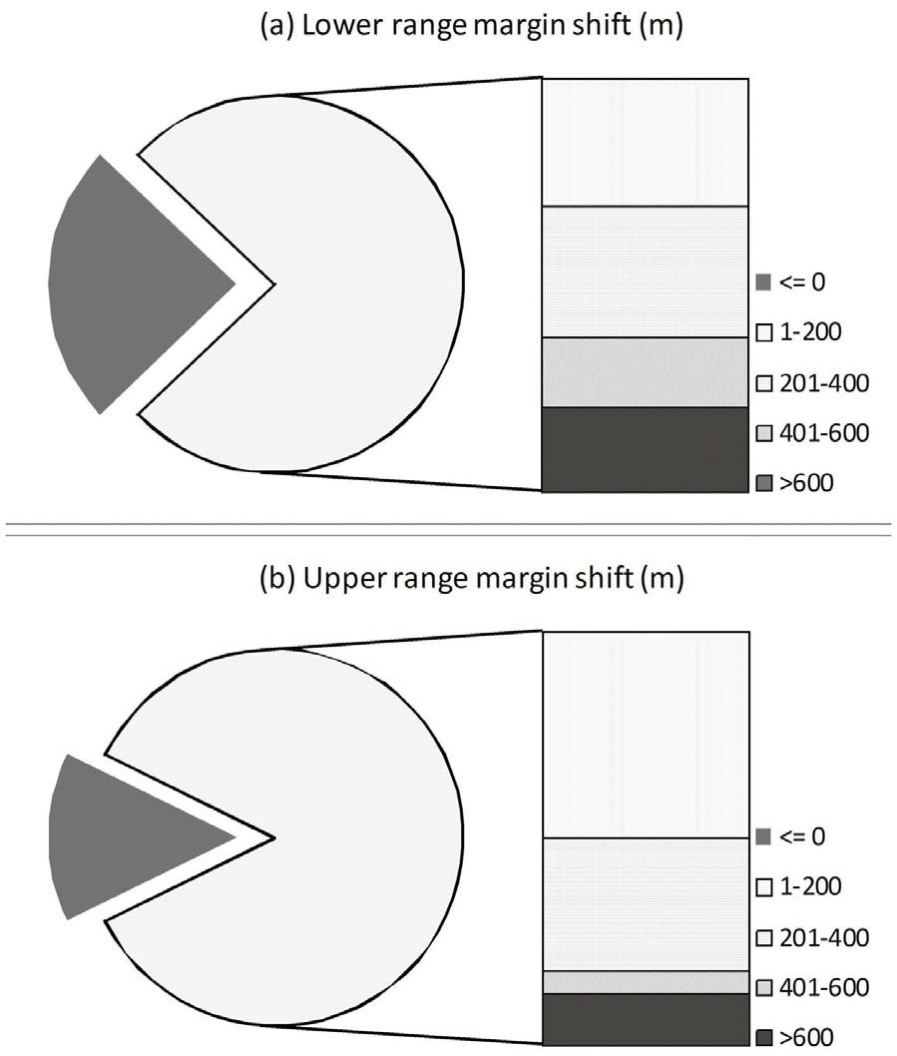
Magnitude of shift (m) in elevational range margins of alpine species in Lhonakh and Lachen valleys in Sikkim during the last century. (A) Shift in lower elevational range margin. (B) Shift in upper elevational range margin. Dark grey portion of the pie chart represents proportion of species with no upward shift in upper or lower range margins, whereas light grey represents species showing upward shifts in upper or lower range margins. Note that significant upward shifts were observed in lower range margins of the species.

We found that nearly 90% endemic plant species of alpine Sikkim Himalaya, having wide taxonomic affiliations, shifted their elevational range margins. The median upward shift in the 124 endemic species’ ranges was 240 m (95% confidence interval: 202.4, 279.5) (Figure 2). More than half of the species studied exhibited an upward shift in the range of 100–400 m (50.8%; 95% CI = 41.9, 59.7). Nearly 12% of these endemic species shifted over 600 m above their known historical ranges (95% CI = 6.5, 17.7) (Figure 3). The upward shift in their lower range margin was 265.4 m (95% CI = 213.9, 320.0), and 214.4 m for their upper margin (95% CI = 174.5, 256.7).

**Figure 4:**
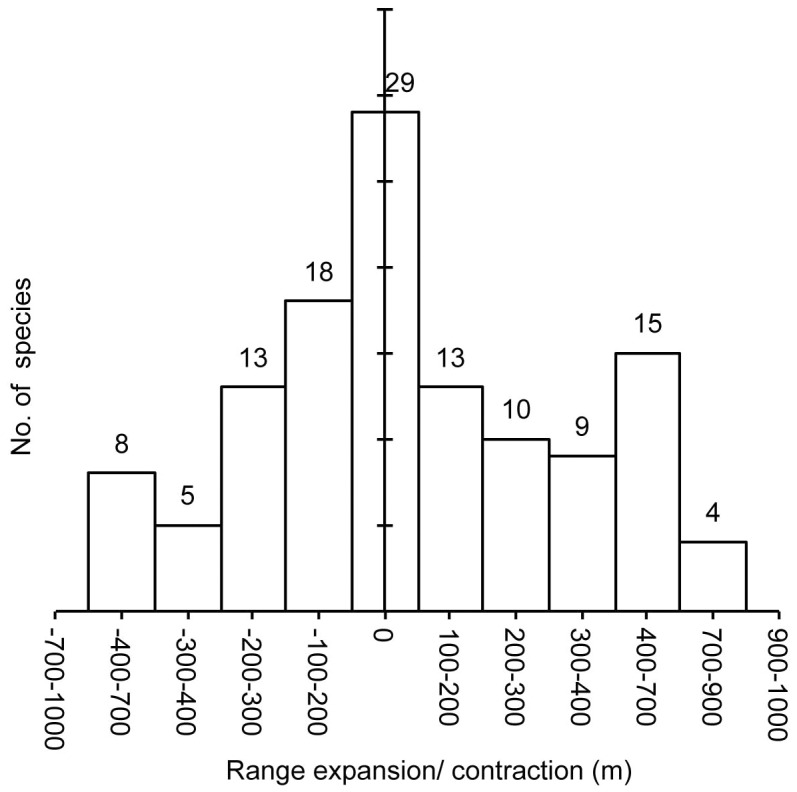
Magnitude of range expansions and contractions in alpine species of Sikkim Himalaya. The difference in lower and upper range margins for a species is taken as its range extent. ‘0′ represents no changes between historic and recent range extents, ‘+’ represents positive changes in historic and recent range extents i.e., range expansion, whereas ‘−’ represents range contraction between historic and recent range extents.

The observed upward shifts in the species’ range margins were categorized into four response types: (i) species in which both upper and lower margins shifted (52%), (ii) species in which only the lower margin shifted (19%), (iii) species in which only the upper margin shifted (20%), and (iv) species in which neither upper nor lower range margins shifted (9%). The species of the lower elevational band of alpine region (4000–4500 m) showed more pronounced upward shifts compared to the species of higher elevational bands (>4700 m). Notably, all the plant species with the largest range shift of 600–800 m were from the lower elevational band of the alpine zone (4000–4500 m). More than 50% of species showed an overall range expansion of 100–300 m in extent, while 62% species showed range contraction of 100–300 m (Figure 4).

There were 6 species (*Rheum nobile* Hook.f. & Thomson, *Saussurea stella* Maxiim., *Rhodiola bupleuroides* Wallich exi Hook.f. & Thomson, *Saussurea uniflora* Wall., *Saussurea leontodontoides* (DC.), *Ponerorchis chusua* (D. Don) Soo.) that showed range contraction by more than 50% of their recorded historical range extents, while 4 species (*Microgynaecium tibeticum* Hook.f., *Meconopsis simplicifolia* Walp., *Pedicularis trichoglossa* Hook. f. and *Lancea tibetica* Hook. f. & Thomson) exhibited range expansion by more than 100% of their historical range extents.

### Species Richness

**Figure 5:**
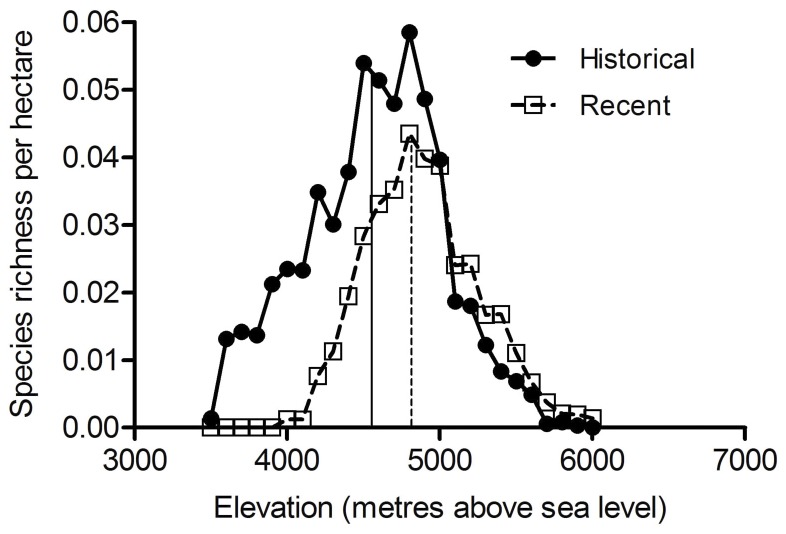
Comparative species richness patterns of endemic alpine species. The recent and historical patterns are shown by elevation, scaled by available habitat area (summed across 100 m elevational bins). The vertical lines represent the 50% cumulative species count for historical (solid) and recent (dashed) surveys.

**Figure 6:**
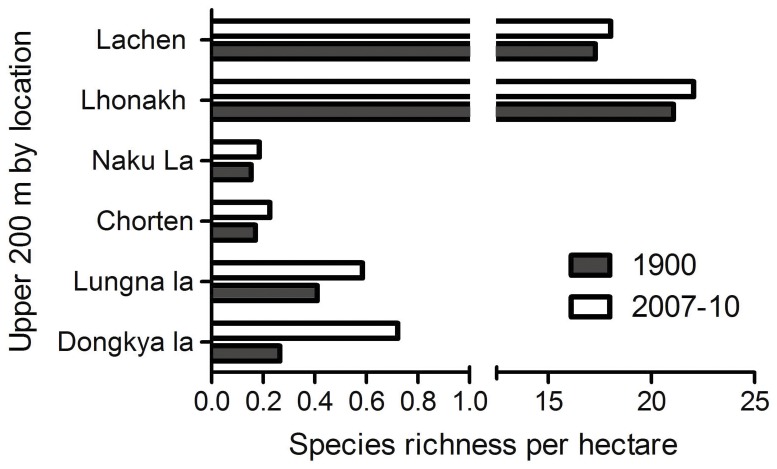
The increase in number of species at the uppermost 200 m elevations of the mountain passes during the last century, scaled by available habitat area.

Richness patterns of the studied species showed a mid-domain peak during both the historical and recent time periods. However, the species richness maxima (calculated as the elevation at which 50% of the cumulative species counts per hectare occurred) shifted upwards by 259 m, from 4548 m in the historical records to 4807 m in the recent investigations, and overall species richness per ha declined by 37% (Figure 5). These investigations also revealed that the number of species at the uppermost 200 m altitudinal band of the four investigated mountain passes have increased compared to the historical records, with mean species richness increasing by 14.7 (range: 3–31) (Figure 6). In the historic records average 8.5 species (range: 3–17) were reported from these passes. However, recent surveys showed 15–18 new species in Lachen and Lhonakh valleys which had not been reported in the historical surveys, suggesting new arrivals (Figure 5).

## Discussion

Adjustments to elevational ranges or migration of plant species into alpine areas occurs in order to occupy more suitable habitats and retreat from less viable areas under the stressor of climatic warming [37]. These changes are reflected either in the upward shifts of the range margins [38] or increase in species richness at mountain peaks [34], [39]. In this study we documented pronounced upward shifts in species’ ranges across the endemic species and plant families studied. Our results seem consistent with the observed upward temperature trends of the study area and the Himalayan region as a whole [16]. An increase in the mean temperature of the coldest months by over 3°C and a decrease in the diurnal temperature range by 1–3°C over the past century was observed in the present study. The critical determinant of elevational shift is the recorded increase in the minimum temperatures of about twice that of maximum temperatures in the high mountains (see [40]).

We observed a shift of 23–998 m in species’ upper elevation limit as compared to that of 30–430 m in the Alps [35]. Also, mean upward displacement rates/decade in the present study was 27.53±22.04 as compared to 34.2±26.7 to 27.8±14.6 m/decade recorded in the Alps [40]. Our results, compared to the studies in the European Alps, indicate higher species’ elevational range shift but similar overall upward species displacement rates/decade. The upward shift of the species reported here is mostly temperature-driven, therefore, our findings are consistent with those from other mountain ranges [35]. However, besides global drivers, a variety of regional factors such as land-use changes, deforestation and heat island effect may likely play important role in the Himalayan warming [24], [26]. More focused studies are needed to understand the impact of human activities on the regional Himalayan climate change.

Our analysis showed that the endemic species of this region are likely to have had different distributional ranges in 1849–1850 and that the distribution was determined by the prevailing cooler climate in the region. A relatively cold and wet climate for this region was reported in the period between 1750 and the early 19^th^ century [41]. The earth’s global mean climate warmed by nearly 0.6°C during the periods between 1910 and 1945, and from 1976 onwards the biological responses of warming have become increasingly manifest [42]. In the present study, the patterns of upward shift in alpine plant species due to climate warming were identified across the three elevational bands with different dominant communities: dwarf scrubs (4000–4500 m), the alpine meadows (4500–4800 m), and the alpine steppes (4800–5500 m). We found that nearly three-fourths of the endemic species from the dwarf-scrub communities at the lower elevational band (4000–4500 m) exhibited an average upward shift of 300 m. The shifts in species of alpine meadows and steppes, occupying higher elevations (4500–5500 m), were not as pronounced. These results appear inconsistent with the comparable warming trends observed at various elevational bands. The temperature changes have been steeper at higher elevational bands in the Himalaya, which have not been followed by commensurate northward elevational species’ range extensions. This is likely due to differences in the prevailing environmental conditions and physiologically available habitats at the two elevational bands (including edaphic conditions, limitations on growth periods, and habitat suitability).

Although sizeable habitable areas exists in the high-altitude Lhonakh Valley (30,520 ha above 5,500 m), it is unclear whether many Himalayan alpine plant species will be able to shift northwards at sufficient pace during the 21^st^ century. Indeed, the smaller shifts observed in the upper range margins (Figure 3) and the declining species richness (especially at higher altitudes, see Figure 5) is a serious challenge that requires continuous monitoring. For Lachen Valley, only 127 ha of habitable area exists above 5,000 m and yet 31 species have already extended their upper range margins to this zone. With no more habitats available further northward, these species will likely suffer from compression of range extents. If the climate warming trend continues these species may become locally extinct [43].

The wide taxonomic range of the species exhibiting altitudinal shifts suggests that there are no phylogenetic confounding factors or clustering, but instead a common environmental variable contributing to the migration. Temperature being the main controlling factor of alpine plant life, our investigations suggest that the species range shifts across phylogenetic affiliations are a direct result of warming [39], [44].

Modelling studies have shown that species at lower elevations (sub-alpine) have a wider range tolerance and more availability of suitable habitats [45]. These observations are consistent with our findings that the elevational range shift of lower band alpine species (4000–4500 m) are more pronounced compared to species of upper elevational bands. It is also likely that the species of lower alpine zone have better adaptability to colonize new habitats as opposed to the species at higher elevational zone. As a result, the failure to adapt at higher elevational zones may result in exacerbated species extinction.

Moreover, there are reports of 5–15% increase in monsoon precipitation in the Himalaya which is affected by global warming (see [46], [47]). The current average annual precipitation over Sikkim, as interpolated from Worldclim dataset [3], was 768.5 mm, but on a year-to-year basis it varied from 0 to 3150 mm. The precipitation levels in Sikkim are projected to increase by 4.1% by 2050 and 8.9% by 2080 which in combination with early snow melt is likely to result in varied hydrological regimes of the soil. This altered edaphic condition is likely to favor the expansion of alpine meadows, thereby shrinking the steppes as compared to their present distribution in higher reaches.

Yet another factor that may influence species range extensions in the alpine regions could be grazing. A recent study in the in the Western Himalaya showed that grazing patterns can influence the vegetation of the area [48].This observation may suggest that our results could be partly influenced by grazing. However, our study area is vastly different from the one referred to above [48] as far as use of alpine areas for pasture land is concerned. The alpine areas in the Western Himalaya, where the above cited study was conducted, have been traditionally used by shepherds and local people as ‘bugyals’ or alpine pastures for grazing and there is recorded history to this effect. In Sikkim, however, sheep rearing is not as prevalent as in the Western Himalaya and as mentioned earlier, we did not encounter such biotic pressures in our study area.

### Conclusion

Considering the overall Himalayan biological diversity scenario we find that the ecosystems at lower elevations in tropical, sub-tropical and temperate zones are subjected to varied land-use/land-cover changes driven by deforestation for agriculture, urbanization, and in the recent past, by unprecedented hydropower development [26], [49]. The cumulative effect of factors such as increased fragmentation of montane floras because of human activities, topographic isolation and endemism along with effects of climate change could result in escalation of extinction rates.

## Supporting Information

Figure S1 **Mean temperature of the warmest and the coldest months in alpine Sikkim Himalaya.**
(TIF)

Table S1 **List of endemic species recorded from the study area with their historic and recent elevational range extents.**
(DOC)

Table S2 **Recent and historic temperature records at different elevations in alpine Sikkim Himalaya.**
(DOC)
