# Supplementary figures and images for: Climate-Induced Elevational Range Shifts and Increase in Plant Species Richness in a Himalayan Biodiversity Epicentre
Source: PLoS One. 2013 Feb 20;8(2):e57103. doi: 10.1371/journal.pone.0057103 (PMC3577782; doi:10.1371/journal.pone.0057103)

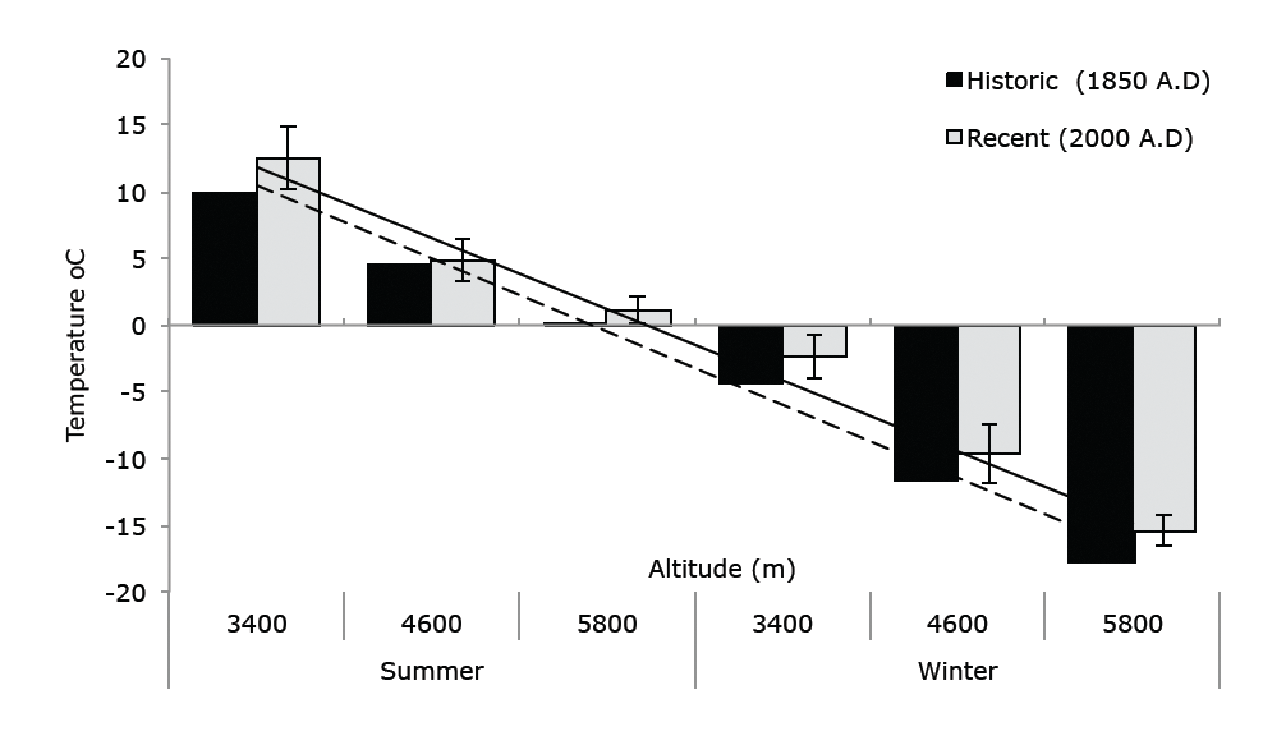

Supplement: Figure S1 — Mean temperature of the warmest and the coldest months in alpine Sikkim Himalaya. (TIF) [file pone.0057103.s001.tif]
