# Supplementary material for: Climate-Induced Elevational Range Shifts and Increase in Plant Species Richness in a Himalayan Biodiversity Epicentre
Source: PLoS One. 2013 Feb 20;8(2):e57103. doi: 10.1371/journal.pone.0057103 (PMC3577782; doi:10.1371/journal.pone.0057103)
Supplement: Table S1 — List of endemic species recorded from the study area with their historic and recent elevational range extents. (DOC) [file pone.0057103.s002.doc]

Table S1. List of the endemic species recorded from the study area with the historic and recent altitudinal range extents.

| **S.No.** | **Species** | **Family** | **Historical range** | **Present range** |
| --- | --- | --- | --- | --- |
| 1 | *Aconogonon hookeri* | Polygonaceae | 4272-5181 | 4300-5250 |
| 2 | *Allium macranthum* Baker. | Alliaceae | 3500-4500 | 3917-4500 |
| 3 | *Allium sikkimense* Baker | Alliaceae | 3886-4420 | 4478-5223 |
| 4 | *Anaphalis xylorhiza* Schultz-Bip | Asteraceae | 3656-5182 | 4000-5195 |
| 5 | *Androsace selago* Klatt. | Primulaceae | 4267-4572 | 4209-4570 |
| 6 | *Anemone demissa* Hook. f. & Thomson | Ranunculaceae | 3963-4877 | 4000-5000 |
| 7 | *Arenaria ciliolata* Edgew. | Caryophyllaceae | 4267-5182 | 4200-5200 |
| 8 | *Arenaria densissima* Wall. | Caryophyllaceae | 4572-5182 | 4500-5286 |
| 9 | *Arenaria glanduligera* Edgew | Caryophyllaceae | 4267-5334 | 4504-5416 |
| 10 | *Arenaria monticola* | Caryophyllaceae | 4572-4877 | 4656-5552 |
| 11 | *Arenaria melandroyoides* Edgew. | Caryophyllaceae | 4267-5334 | 4648-5400 |
| 12 | *Arenaria musciformis* Wall. | Caryophyllaceae | 4420-5029 | 4384-5186 |
| 13 | *Arenaria polytrichoides* Edgew | Caryophyllaceae | 4267-5182 | 4492-5396 |
| 14 | *Artemisia biennis* Willd. | Asteraceae | 4572-4877 | 4500-5000 |
| 15 | *Artemisia campbelli* Hook. f. & Thomson | Asteraceae | 3658-4722 | 3670-4877 |
| 16 | *Artemsia salsoloides* Willd. | Asteraceae | 4572-5182 | 4500-5200 |
| 17 | *Aster diplostephoides* Benth. | Asteraceae | 4267-4877 | 4315-5093 |
| 18 | *Astragalus confertus* Benth | Leguminosae | 4267-4877 | 4548-5248 |
| 19 | *Berberis angulosa* Wall. | Berberidaceae | 3658-4572 | 3828-4772 |
| 20 | *Berberis concinna* Hook. f. & Thomson | Berberidaceae | 3810-4191 | 4089-4778 |
| 21 | *Caltha scaposa* Hook.f. | Ranunculaceae | 4267-5182 | 4200-5350 |
| 22 | *Campanula aristata* Wall. | Campanulaceae | 3957-4724 | 4328-4907 |
| 23 | *Campanula immodesta* Hook. f. & Thomson | Campanulaceae | 3963-4725 | 4094-4975 |
| 24 | *Cassiope fastigata* (Wallich).D.Don. | Ericaceae | 3048-4267 | 3708-4508 |
| 25 | *Cassiope selaginoides* Hook. f. &Thomson | Ericaceae | 3048-3962 | 3825-4789 |
| 26 | *Chamaesium novem-jugum* | Apiaceae | 3658-4572 | 3700-4902 |
| 27 | *Chionocharis hookeri* (C. B. Clarke) I. M. Johnston | Asteraceae | 4662-5393 | 4680-5395 |
| 28 | *Codonopsis foetens* Hook. f. & Thomson | Campanulaceae | 4115-4648 | 4315-4822 |
| 29 | *Codonopsis thalictrifolia* Wall. | Campanulaceae | 3962-4420 | 4000-4700 |
| 30 | *Cortia hookeri Clarke* | Apiaceae | 3962-5182 | 3950-5500 |
| 31 | *Corydalis cashmeriana* Royle. | Papaveraceae | 4267-5182 | 4700-5240 |
| 32 | *Cotoneaster microphylla* Wall. | Rosaceae | 3352-4572 | 3700-4572 |
| 33 | *Cremanthodium decaisnei* Clarke | Asteraceae | 4420-4877 | 4397-5057 |
| 34 | *Cremanthodium oblongatum* Clarke | Asteraceae | 3658-4877 | 3800-4880 |
| 35 | *Cremanthodium reniforme* Benth | Asteraceae | 3048-4572 | 3785-4572 |
| 36 | *Cyananthus incanus* Hook. f. & Thomson | Campanulaceae | 3963-4877 | 3988-5125 |
| 37 | *Delphinium caeruleum* Jacq. | Ranunculaceae | 4710-5182 | 4850-5268 |
| 38 | *Delphinium glaciale* Hook.f. | Ranunculaceae | 4267-4877 | 4300-5050 |
| 39 | *Dracocephalum heterophyllum* Benth | Lamiaceae | 4500-4877 | 4789-5192 |
| 40 | *Elsholtzia eriostachya* Benth. | Lamiaceae | 4000-4420 | 4100-4880 |
| 41 | *Eritrichium pustulosum* C. B. Clarke | Boraginaceae | 3963-5182 | 4412-5192 |
| 42 | *Eritrichium pygmaeum* Clarke | Boraginaceae | 4267-4750 | 4207-4750 |
| 43 | *Erysimum deflexum* Hook. f. & Thomson | Brassicaceae | 4267-5259 | 4330-5259 |
| 44 | *Euphorbia stracheyi* Boiss | Euphorbiaceae | 3658-4877 | 3873-5520 |
| 45 | *Euphrasia officinalis* Linn. | Scrophulariaceae | 3000-4265 | 3887-4600 |
| 46 | *Fragaria daltoniana* J. Gay | Rosaceae | 3048-4572 | 3664-4649 |
| 47 | *Gaultheria trichophylla* Royle. | Ericaceae | 3352-4267 | 3700-4649 |
| 48 | *Gentiana detonsa* Fries | Gentianaceae | 3962-4267 | 4044-4951 |
| 49 | *Gentiana ornata* (G.Don).Griesb | Gentianaceae | 4286-4725 | 4375-4899 |
| 50 | *Gentiana robusta* King. | Gentianaceae | 4267-4877 | 4507-4900 |
| 51 | *Geranium collinum* M.Bieb. | Geraniaceae | 3658-4268 | 4100-4556 |
| 52 | *Hedysarum sikkimense* Benth. | Leguminosae | 3962-4876 | 4552-5016 |
| 53 | *Hippolytia gossypina* Hook. f. & Thomson | Asteraceae | 4419-5182 | 4500-5250 |
| 54 | *Juniperus indica* Bertol | Coniferae | 4000-4572 | 3822-4600 |
| 55 | *Juniperus recurva* Ham. | Coniferae | 4000-4572 | 3955-4649 |
| 56 | *Lagotis glauca* Gaertn. | Selaginaceae | 4267-4572 | 4300-4900 |
| 57 | *^#^Lancea tibetica* Hook. f. & Thomson | Scrophulariaceae | 4419-4572 | 4350-4956 |
| 58 | *Leontopodium haastioides* Hand.-Mazz | Asteraceae | 4420-5182 | 4645-5418 |
| 59 | *Lepidium capitatum* Hook.f.&Thomson | Brassicaceae | 4200-5486 | 4500-5552 |
| 60 | *Lonicera hispida* Poll. | Caprifoliaceae | 3962-5182 | 4000-5000 |
| 61 | *Meconopsis horridula* Hook. f. & Thomson | Papaveraceae | 4420-5029 | 4495-5351 |
| 62 | *^#^Meconopsis simplicifolia* Walp*.* | Papaveraceae | 3500-3658 | 3850-4656 |
| 63 | *^#^Microgynaecium tibeticum* Hook.f. | Brassicaceae | 4500-4562 | 4500-4956 |
| 64 | *Microula pustulosa* (C. B. Clarke) Duthie | Boraginaceae | 3962-5182 | 4207-5220 |
| 65 | *Morina nepalensis* D. Don. | Morinaceae | 3048-3968 | 3700-4594 |
| 66 | *Nardostachys grandiflora* DC. | Valerianaceae | 3981-5182 | 4030-5054 |
| 67 | *Nepeta discolor* Royle. ex Bentham | Lamiaceae | 3963-4876 | 4500-5000 |
| 68 | *Onosoma hookeri* Clarke | Boraginaceae | 4115-4572 | 4136-4825 |
| 69 | *Oreosolen wattii* Hook.f. | Scrophulariaceae | 4572-5030 | 4500-5000 |
| 70 | *Oxytropis tartarica* Jacq. | Leguminoseae | 4000-4572 | 4507-4916 |
| 71 | *Parnassia nubicola* Wall. | Scrophulariaceae | 3524-4572 | 4150-4879 |
| 72 | *Pedicularis integrifolia* Hook.f. | Scrophulariaceae | 3657-4344 | 3978-5086 |
| 73 | *Pedicularis lachnoglossa* Hook.f. | Scrophulariaceae | 3657-4510 | 3475-4500 |
| 74 | *Pedicularis longifolia* Rudolph | Scrophulariaceae | 4420-4877 | 4507-4940 |
| 75 | *Pedicularis roylei* Maxim. | Scrophulariaceae | 4562-4877 | 4507-4916 |
| 76 | *^#^Pedicularis trichoglossa* Hook. f. | Scrophulariaceae | 4572-4752 | 4500-4700 |
| 77 | *Phlomis rotata* Bentham ex J.D. Hooker | Lamiaceae | 3963-4876 | 4508-5552 |
| 78 | *Polygonum sibiricum* Laxm. | Polygonaceae | 4420-5182 | 4745-5211 |
| 79 | **Ponerorchis chusua* (D. Don) Soo. | Orchidaceae | 3048-4114* | 4000-4522* |
| 80 | *Potentilla fruticosa* | Rosaceae | 3352-5182 | 4209-5250 |
| 81 | *Potentilla microphylla* | Rosaceae | 4267-4572 | 4507-4916 |
| 82 | *Potentilla sino-nivea* | Rosaceae | 4267-5334 | 4500-5250 |
| 83 | *Primula sikkimensis* Hook.f. | Primulaceae | 3352-4572 | 4000-4865 |
| 84 | *Primula tibetica* Watt | Primulaceae | 4496-5182 | 4438-5400 |
| 85 | *Pterocephalus hookeri* (C. B. Clarke) Diels | Dipsacaceae | 4267-4876 | 4507-5248 |
| 86 | **Rheum nobile* Hook.f. & Thomson | Polygonaceae | 3962-4572* | 4556-4656* |
| 87 | *Rheum spiciforme* Royle | Polygonaceae | 3962-4876 | 4125-4813 |
| 88 | **Rhodiola bupleuroides* Wallich ex Hook.f. & Thomson | Crassulaceae | 3200-4542* | 4504-5000* |
| 89 | *Rhododendron nivale* Hook.f. | Ericaceae | 3352-4876 | 3963-5102 |
| 90 | *Ribes luridum* Hook. f. & Thomson | Hydrangeaceae | 3048-4572 | 3770-4772 |
| 91 | *Salix calyculata* Hook.f. | Salicaeae | 3352-4267 | 4200-5000 |
| 92 | *Salix lindlyeana* Wall. | Salicaeae | 3658-4267 | 4000-4500 |
| 93 | *Saussurea gossypiphora* Don. | Asteraceae | 4267-5182 | 4606-5000 |
| 94 | *Saussurea heiracioides* Hook.f | Asteraceae | 3657-4267 | 4485-4836 |
| 95 | *Saussurea katochaete* | Asteraceae | 3963-4877 | 4050-5560 |
| 96 | **Saussurea leontodontoides* (DC.) | Asteraceae | 3962-5182* | 4540-5129* |
| 97 | **Saussurea stella* Maxim. | Asteraceae | 4445-4876* | 4725-4880* |
| 98 | *Saussurea tridactyla* Clarke | Asteraceae | 4740-5334 | 4919-5315 |
| 99 | **Saussurea uniflora* Wall. | Asteraceae | 3048-4572* | 4256-4902* |
| 100 | *Saussurea werneroides* Hook.f. | Asteraceae | 4724-5129 | 4700-5200 |
| 101 | *Saxifraga aristulata* Hook. f. & Thomson | Saxifragaceae | 3962-5182 | 4438-5248 |
| 102 | *Saxifraga flagellaris* Willd. | Saxifragaceae | 4267-4876 | 4500-5000 |
| 103 | *Saxifraga hemisphaerica* Hook. f. & Thomson | Saxifragaceae | 4876-5182 | 4500-5200 |
| 104 | *Saxifraga hirculus* Linn. | Saxifragaceae | 4267-5182 | 4735-5248 |
| 105 | *Saxifraga jacquemontiana* Dene. | Saxifragaceae | 4267-5029 | 4700-5248 |
| 106 | *Saxifraga lychinits aff.* | Saxifragaceae | 4267-5029 | 4428-5400 |
| 107 | *Saxifraga pallida* Wall. | Saxifragaceae | 3810-5106 | 4250-5220 |
| 108 | *Saxifraga ramulosa* Wall. | Saxifragaceae | 4572-5182 | 4500-5200 |
| 109 | *Saxifraga saginoides* J. D. Hooker & Thomson | Saxifragaceae | 3048-5486 | 3730-5552 |
| 110 | *Sedum fischeri* R.Hamet. | Crassulaceae | 4877-5182 | 4800-5200 |
| 111 | *Sibbaldia purpurea* Hook.f. | Rosaceae | 4267-4877 | 4200-5010 |
| 112 | *Silene apetala* Willd. | Caryophyllaceae | 3962-4572 | 4000-4572 |
| 113 | *Silene caespitella* F. N. Williams | Caryophyllaceae | 3657-4267 | 4485-4902 |
| 114 | *Silene nigrescens* (Edgeworth) Majumdar | Caryophyllaceae | 4572-4876 | 4735-4938 |
| 115 | *Soroseris glomerata* (Decne.) Stebb | Asteraceae | 3658-4268 | 4248-4926 |
| 116 | *Stellaria decumbens* Edgew. | Caryophyllaceae | 3962-5182 | 4248-5248 |
| 117 | *Stracheya tibetica* Benth. | Leguminosae | 4267-5182 | 4570-5348 |
| 118 | *Swertia multicaulis* Don. | Gentianaceae | 4267-4572 | 4570-4900 |
| 119 | *Thalictrum alpinum* Linn. | Ranunculaceae | 4267-4876 | 4507-5250 |
| 120 | *Thlaspi alpestre* Linn. | Brassicaceae | 4562-5171 | 4700-5240 |
| 121 | *Viola biflora* Linn. | Violaceae | 4000-4876 | 4200-5248 |
| 122 | *Waldheimia tridactylites* | Asteraceae | 4511-5133 | 4500-5129 |
| 123 | *Youngia depressa* Hook. f. & Thomson | Asteraceae | 4572-4877 | 4507-4916 |
| 124 | *Youngia gracileps* Hook.f. | Asteraceae | 4572-4877 | 4507-4965 |

*These species recorded range contraction by more than 50 % of their recorded historical range extents.

*^#^*These species exhibited range expansion by more than 100 % of their former range extents.
