# Supplementary material for: Climate-Induced Elevational Range Shifts and Increase in Plant Species Richness in a Himalayan Biodiversity Epicentre
Source: PLoS One. 2013 Feb 20;8(2):e57103. doi: 10.1371/journal.pone.0057103 (PMC3577782; doi:10.1371/journal.pone.0057103)
Supplement: Table S2 — Recent and historic temperature records at different elevations in alpine Sikkim Himalaya. (DOC) [file pone.0057103.s003.doc]

*Table S2. Recent and historic temperature records at different elevations in alpine Sikkim Himalaya.*

| **2007-2010** | | **1848-1849** | |
| --- | --- | --- | --- |
| Alt (m) | Temp (° C) | Alt (m) | Temp (° C) |
| **Mean daily temperature range (°C)** | | | |
| 3200-3500 | 8.93 ± 2.2 | 3352 | 11 |
| 4400-4600 | 7.9 ± 3.0 | 4572 | 15 |
| 5300-5555 | 18.21 ± 0.2 | 5791 | 19 |
| **Mean warmest month (°C)** | | | |
| 3200-3500 | 11 ± 4.3 | 3352 | 10 |
| 4400-4600 | 4.9 ± 1.6 | 4572 | 4.44 |
| 5300-5555 | 0.8 ± 0.97 | 5791 | 0 |
| **Mean coldest month (°C)** | | | |
| 3200-3500 | -2.83 ± 5.6 | 3352 | -4.44 |
| 4400-4600 | -7.92 ± 3 | 4572 | -11.67 |
| 5300-5555 | -12.17 ± 1.2 | 5791 | -17.78 |
| **Temperature lapse rate for a 1 °C decrease with increasing altitude (m)** | | | |
| 3200-3500 | 120 | 3352 | 98 |
| 4400-4600 | 250 | 4572 | 107 |
| 5300-5555 | 204 | 5791 | 121 |
